# Efficient Tissue Detection in Whole-Slide Images Using Classical and Hybrid Methods: Benchmark on TCGA Cancer Cohorts

**DOI:** 10.3390/cancers17172918

**Published:** 2025-09-05

**Authors:** Bogdan Ceachi, Filip Muresan, Mihai Trascau, Adina Magda Florea

**Affiliations:** 1 Faculty of Automatic Control and Computers, National University of Science and Technology POLITEHNICA Bucharest, 060042 Bucharest, Romania; mihai.trascau@upb.ro (M.T.); adina.florea@upb.ro (A.M.F.); 2 Victor Babeş National Institute of Research and Development in Pathology & Biomedical Sciences, Carol Davila University of Medicine and Pharmacy, 050474 Bucharest, Romania; filip-cristian.muresan@drd.umfcd.ro

**Keywords:** whole-slide imaging, tissue detection, cancer histopathology, TCGA cohorts, machine learning, annotation-free methods, computational pathology

## Abstract

**Background**: Whole-slide images (WSIs) are crucial in pathology for digitizing tissue slides, enabling pathologists and AI models to analyze cancer patterns at gigapixel scale. However, their large size incorporates artifacts and non-tissue regions that slow AI processing, consume resources, and introduce errors like false positives. Tissue detection serves as the essential first step in WSI pipelines to focus on relevant areas, but deep learning detection methods require extensive manual annotations. Methods: This study benchmarks four thumbnail-level tissue detection methods—Otsu’s thresholding, K-Means clustering, our novel annotation-free Double-Pass hybrid, and GrandQC’s UNet++ on 3322 TCGA WSIs from nine cancer cohorts, evaluating accuracy, speed, and efficiency. **Results:** Double-Pass achieved an mIoU of 0.826—very close to the deep learning GrandQC model’s 0.871—while processing slides on a CPU in just 0.203 s per slide, markedly faster than GrandQC’s 2.431 s per slide on the same hardware. As an annotation-free, CPU-optimized method, it therefore enables efficient, scalable thumbnail-level tissue detection on standard workstations. **Conclusions:** The scalable, annotation-free Double-Pass pipeline reduces computational bottlenecks and facilitates high-throughput WSI preprocessing, enabling faster and more cost-effective integration of AI into clinical pathology and research workflows. Comparing Double-Pass against established methods, this benchmark demonstrates its novelty as a fast, robust and annotation-free alternative to supervised methods.

## Simple Summary

A crucial quality-control step in digital pathology is to identify tissue regions within a whole-slide image before deciding where AI models should operate. In this study, we introduce Double-Pass, a new annotation-free method that runs on standard CPUs and nearly matches the performance of a fully supervised UNet++ on 3322 annotated TCGA slides (mIoU 0.826 vs. 0.871) while processing each slide in just 0.20 s compared with the UNet++ model’s 2.43 s CPU inference. We also compared Double-Pass against classical Otsu and K-means methods to demonstrate its advantages. By providing a fast, label-free quality-control step, Double-Pass ensures that subsequent AI models operate only on relevant tissue regions without the burden of manual annotation.

## 1. Introduction

Tissue detection is the critical first step in WSI pipelines, creating a mask to focus processing on relevant areas. In cancer research, this is especially vital due to heterogeneous staining (e.g., faint areas in necrotic tumors) and variability across scanners. Traditional methods are fast but fail on subtle tissues, while deep learning excels but demands annotated data—a challenge in digital pathology, where expert labeling is time-consuming and scarce for diverse cancers. Annotation burdens can delay projects, particularly in rare cancers where data is limited.

In this work, we introduce Double-Pass, a novel annotation-free hybrid method for tissue detection in WSIs, which combines two classical yet complementary strategies to enhance robustness while maintaining CPU-level efficiency. Unlike deep learning methods that require extensive annotations and GPU resources, Double-Pass is entirely unsupervised and achieves performance close to state-of-the-art models such as GrandQC’s UNet++. We benchmark our approach alongside three other methods: Otsu thresholding, K-Means clustering, and GrandQC, on 3322 annotated TCGA WSIs across nine cancer cohorts. Compared to prior tissue-detection benchmarks, our study evaluates a broader range of cancer types using publicly available tissue-versus-background masks released by GrandQC [1]. This design ensures that Double-Pass and other methods are evaluated on the same diverse dataset, highlighting their robustness and reproducibility across different cancers. By emphasizing accuracy, inference time, and resource efficiency, this study provides a comprehensive comparison and positions Double-Pass as a practical and scalable tool for preprocessing in digital pathology and cancer AI pipelines.

## 2. Literature Review

Tissue detection methods applied to WSIs images are essential for digital pathology workflows. They filter irrelevant regions to streamline AI analyses for any objective by training the model on multiple cancer types (like breast cancer, bladder cancer, etc). Classical methods, such as Otsu’s thresholding [2], are fast and annotation-free but struggle with cancer-specific challenges like variable staining in heterogeneous tumors. Song et al.’s EntropyMasker [3] improves on this for porous tissues, demonstrating higher sensitivity and Jaccard scores, but its performance on faint cancer edges remains limited. Chen and Yang’s tissueloc [4] combines grayscale thresholding with morphological operations for rapid localization, which is ideal for resource-constrained digital pathology labs.

Deep learning models offer superior robustness. Bándi et al.’s resolution-agnostic CNN [5] segments tissue across stains and scanners, generalizing well to diverse cancer types. Weng et al.’s GrandQC UNet++ [1] excels in multi-class artifact detection across TCGA, providing high precision for preprocessing in AI-driven cancer analyses. Wang et al. [6] applied CNNs for metastatic breast cancer detection, underscoring the need for accurate tissue masks to avoid false positives in lymph nodes.

Cancer-focused tools like TIAToolbox [7] integrate detection into end-to-end pipelines, yet annotation costs limit scalability. Foundation models like Phikon-v2 [8] and Virchow [9] pre-filter non-tissue, but their deep approaches demand resources. Hybrid methods, like our Double-Pass method, address this challenge by combining classical speed with robustness, without annotations, making them suitable for TCGA-like cancer datasets.

Specific pathology applications further illustrate the reliance on tissue detection as a critical first step. For instance, Ceachi et al. [10] developed an AI-based method for automatic identification of lymphovascular invasion in urothelial carcinomas, where initial tissue detection is essential to isolate relevant regions (e.g., tumor nests and vessels) from background artifacts in WSIs, enabling focused segmentation and reducing false positives in invasion detection. Similarly, Zurac et al. [11] proposed an AI approach for detecting Mycobacterium tuberculosis in Ziehl–Neelsen-stained tissue slides, relying on tissue detection to filter out non-tissue areas and artifacts, thus concentrating computational efforts on stained regions for accurate pathogen identification in tuberculosis diagnostics. These studies highlight how tissue detection underpins AI workflows in diagnosing oncological and infectious diseases, ensuring efficiency and precision in the WSI’s downstream analyses.

Several studies have benchmarked tissue segmentation approaches, providing valuable comparisons. For instance, Bándi et al. [12] compared traditional methods like Foreground Extraction from Structure Information (FESI) with fully convolutional networks (FCNNs) and U-Net architectures on 54 WSIs from various tissues and stains, showing DL’s superior accuracy (Jaccard  0.93 vs. 0.68 for traditional). Similarly, Riasatian et al. [13] evaluated U-Net variants with different backbones for background removal in histopathology images, achieving high performance but requiring annotations and focusing on smaller patches rather than whole-slide benchmarks.

Post-processing techniques have been explored to enhance both classical and DL methods. Marczyk et al. [14] proposed morphological post-processing to refine initial segmentations from thresholding or DL on 197 annotated WSIs, significantly improving accuracy.

Large-scale datasets are crucial for robust benchmarking. Nechaev et al. [15] introduced HISTAI, an open-source dataset with over 60,000 WSIs across diverse tissues, stains, and clinical metadata, supporting tasks like diagnostic modeling. Unlike TCGA’s cancer focus, HISTAI covers broader pathology, but both enable large-scale evaluations.

Quality control (QC) pipelines are incorporating segmentation at an increasing rate. Patil et al. [16] developed a semantic segmentation model for QC, identifying tissue, artifacts, and regions in WSIs, similar to GrandQC but emphasizing artifact detection. This aligns with our use of GrandQC as a benchmark, though our study extends to efficiency comparisons across methods.

Emerging foundation models continue to advance the field. Nicke et al. [17] presented Tissue Concepts v2, a supervised foundation model for WSIs, which was pre-trained on histopathology for tasks including segmentation. Unlike self-supervised models like Phikon-v2, its supervised nature may improve task-specific performance, but it still requires labeled data, highlighting the value of a tissue detection method.

These works underscore the evolution from classical to DL and hybrid methods, yet gaps persist in scalable, annotation-free benchmarks on cancer cohorts like TCGA.

## 3. Methods

### 3.1. Dataset and Thumbnail Generation

Our study builds directly on the resources released with the GrandQC project by Weng et al. [1]. The authors curated a heterogeneous training set of H&E-stained whole-slide images (WSIs) drawn from TCGA, all scanned on Leica GT450/AT2/CS2 and Hamamatsu S60/S360 systems at 40× (approximately 0.25 μm ${\mathrm{px}}^{-1}$). Tissue-versus-background masks for these slides were produced semi-automatically in QuPath v0.4.3.

Beyond the training material, GrandQC also open sources quality-control (QC) masks for the entire TCGA archive under a permissive licence. Leveraging this resource, we compiled a benchmark spanning nine diagnostic cohorts: ACC (Adenomas and Adenocarcinomas), BRCA 9 cancer type (Adenomas and Adenocarcinomas, Adnexal and Skin Appendage Neoplasms, Basal Cell Neoplasms, Complex Epithelial Neoplasms, Cystic, Mucinous and Serous Neoplasms, Ductal and Lobular Neoplasms, Epithelial Neoplasms, NOS, Fibroepithelial Neoplasms, Squamous Cell Neoplasms), CESC (Cervical Squamous Cell Carcinoma and Endocervical Adenocarcinoma), CHOL (Cholangiocarcinoma), DLBC (Lymphoid Neoplasm Diffuse Large B-cell Lymphoma), ESCA (Esophageal Carcinoma), GBM (Gliomas), HNSC (Head and Neck Squamous Cell Carcinoma) and LIHC (Adenomas and Adenocarcinomas forliver hepatocellular carcinoma). Exact number of slides per cohort: ACC (206), BRCA (1100), CESC (279), CHOL (39), DLBC (42), ESCA (155), GBM (860), HNSC (274), LIHC (367), totaling 3322 slides.

Table 1 details the cancer types, and organs present in the dataset we used from TCGA.

For every slide, we generate an RGB thumbnail at 10 μm ${\mathrm{px}}^{-1}$ (1× objective), matching the native resolution at which the GrandQC tissue detector operates. Working at 10 μm ${\mathrm{px}}^{-1}$ trades single-cell resolution for faster processing by down-sampling the WSIs to a size sufficient for tissue-versus-background segmentation. At this scale, the thumbnails no longer capture individual cell morphology, but they still preserve tissue boundaries, structural patterns, and gross artefacts needed for reliable tissue detection. This reduction in resolution cuts the data size and I/O by roughly two orders of magnitude, enabling efficient preprocessing while still capturing macro-level cancer features.

### 3.2. Tissue Detector Methods

**Figure 1:**
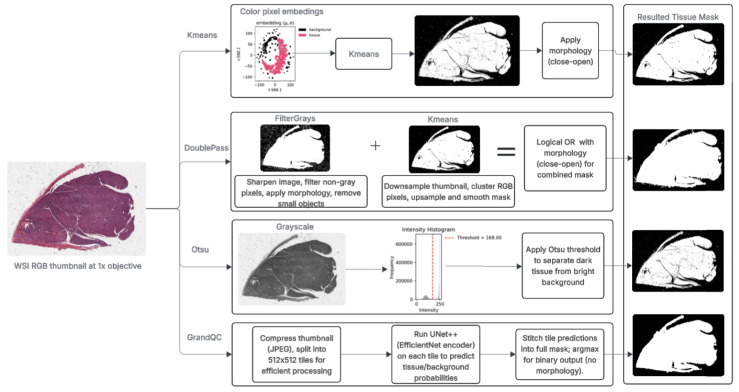
Overview of the tissue detection methods. The flowchart illustrates the key steps for each approach, from input thumbnail to output mask, highlighting preprocessing, segmentation, and post-processing operations. Branches correspond to K-Means (first row), Double-Pass (second row), Otsu (third row), and GrandQC (fourth row), with examples of intermediate outputs for clarity.

Figure 1 provides a visual overview of the four tissue detection pipelines evaluated in this study. Each method processes low-resolution thumbnails (10 μm/px) of whole-slide images (WSIs) to generate binary tissue masks, where tissue pixels are marked as 255 (white) and background as 0 (black). These masks enable focused analysis on relevant regions, which is crucial for efficient processing in large-scale cancer cohorts like TCGA. The methods were selected to represent a spectrum from classical, annotation-free techniques to deep learning, allowing a balanced comparison in terms of accuracy, speed, and resource requirements. Pseudocode for each is detailed in the Appendix A (Algorithms A1–A4), ensuring reproducibility. The figure highlights key steps: color pixel embedding and K-Means clustering (first row), Double-Pass hybrid fusing FilterGrays and downsampled K-Means (second row), Otsu thresholding on grayscale with intensity histogram (third row), and GrandQC’s UNet++ deep learning pipeline fourth row. All operate at thumbnail level to minimize computational load, as full-resolution processing would be infeasible for gigapixel WSIs in oncology workflows.

The pseudocode in the Appendix provides precise implementations, which we discuss here in the context of their operation and suitability for cancer WSIs. For instance, all methods are designed for efficiency on thumbnails, but differ in how they handle color/intensity variations common in H and E-stained cancer tissues.

#### 3.2.1. Global Otsu Thresholding

As shown in the third row of Figure 1, Otsu thresholding [2] begins by converting the RGB thumbnail to grayscale, reducing it to a single-intensity channel (Algorithm A1, Line 2). This simplifies the problem by focusing on brightness differences, where stained tissue typically appears darker than the background. Otsu’s algorithm then computes an intensity histogram and identifies the optimal threshold $T^{*}$ that maximizes between-class variance (Line 3), separating the distribution into tissue and background classes. Pixels below $T^{*}$ are classified as tissue (set to 255 in the mask), while those above are background (set to 0; Line 4).

This method was chosen for its parameter-free nature and sub-second execution on CPUs, making it ideal as a baseline for resource-limited lab digital pathology images. It works by assuming a bimodal intensity distribution, which is often present in H and E-stained slides. Compared to clustering-based methods, Otsu is faster but relies solely on intensity, potentially less robust to variations in staining or artifacts.

#### 3.2.2. Colour-Statistics K-Means Clustering

Illustrated in the top branch of Figure 1, this method clusters pixels using color statistics to capture both intensity and variation (Algorithm A2). The thumbnail is flattened into a pixel list scaled to [0,1] (Line 2), and for each, mean brightness μ (Line 3) and standard deviation σ (Line 4) across RGB channels are computed, forming a 2D feature matrix (Line 5). K-Means partitions these into two clusters (Line 6), selecting the one with lower average μ as tissue (Line 7). The resulting labels form a mask (set to 255 for tissue; Line 8), refined by morphological closing and opening with a 5x5 kernel to smooth edges and remove noise (Line 9).

We selected this approach because it incorporates data texture (σ), in addition to colour intensity, potentially handling variations in staining better than simple thresholding. It works by grouping similar pixels in feature space, adapting to the data distribution. However, processing all pixels can take seconds on large thumbnails. Compared to Otsu, it provides a more feature-rich segmentation but at higher computational cost.

#### 3.2.3. Double-Pass Hybrid Method

The middle branch of Figure 1 depicts Double-Pass, our novel annotation-free hybrid that combines two complementary passes (Algorithm A3). The first pass (FilterGrays) sharpens the thumbnail to enhance edges (Line 2), then marks pixels as tissue if RGB differences exceed 15 levels (Line 3), rejecting uniform grays. Dilation, closing (Line 4), and small-object removal (<5000 pixels; Line 5) refine the mask. The second pass (DownKMeans) downsamples to 25% scale for speed (Line 8), applies K-Means on RGB vectors (Lines 9-11), resizes back (Line 13), and smooths (Line 14). The passes are merged via logical OR (Line 20), followed by final morphology (Line 21).

Double-Pass was developed to combine the strengths of classical methods without requiring annotations or GPUs. It works by fusing color-based artifact rejection with intensity-based detection, aiming for a balanced performance. Compared to single classical methods, it offers improved robustness through hybridization, while remaining efficient on CPUs.

#### 3.2.4. GrandQC UNet++ Model

The bottom-right branch of Figure 1 outlines GrandQC’s UNet++ [1], a deep learning model trained on labeled data for pixel-level prediction (Algorithm A4). The thumbnail is JPEG-compressed (Line 2), tiled into 512x512 patches (Line 3), normalized (Line 5), and fed to UNet++ (Line 6), which uses encoder–decoder architecture with skip connections to learn complex patterns. Probabilities are argmaxed to labels (tissue as 0; Line 7), stitched (Line 8), and converted to a mask (255 for tissue; Line 9).

This serves as a high-accuracy benchmark, chosen for its demonstrated generalization across TCGA data. It works through supervised learning of features from diverse slides. Compared to classical methods, it requires annotations and more resources but can capture intricate patterns.

In summary, classical methods (Otsu, K-Means) prioritize speed and simplicity, Double-Pass balances efficiency and robustness, and GrandQC offers advanced accuracy—tailored choices for oncology-related needs. The pseudocode highlights these differences: e.g., Otsu’s simplicity (few lines) vs. Double-Pass’s multi-pass fusion for better handling of cancer tissue variability.

The Python implementations of these methods, including the novel Double-Pass algorithm, are available in our GitHub repository. Key libraries included scikit-image (v0.21.0), OpenCV (v4.9.0), scikit-learn (v1.5.2, for K-Means), and PyTorch (v2.5.1+cu124, for the GrandQC model). For further details on data and code availability, see the Data Availability Statement.

### 3.3. Evaluation Metrics

To assess the performance of the tissue detection methods, we employ three key metrics: mean Intersection over Union (mIoU), Dice score (also referred to as mean Dice Class Consistency, mDCC), and inference time. These were selected for their relevance to segmentation tasks in imbalanced datasets like WSIs, where tissue regions often occupy a small fraction of the image, and for evaluating practical efficiency in oncology workflows.

The mIoU is defined as $\mathrm{mIoU}=\frac{TP}{TP+FP+FN}$, where TP, FP, and FN represent true positives, false positives, and false negatives, respectively. It measures the overlap between predicted and ground-truth masks, penalizing both over-segmentation (extra tissue detected) and under-segmentation (missed tissue). We chose mIoU because it provides a balanced evaluation of accuracy, which is crucial for avoiding missed tumor regions (false negatives) in cancer analysis.

The Dice score is calculated as $\mathrm{Dice}=\frac{2TP}{2TP+FP+FN}$, emphasizing overlap twice as much as mIoU, making it robust to the class imbalances common in WSIs (e.g., vast background areas). In this study, we use the mean Dice across classes (mDCC) for comprehensive assessment. Dice is selected for its sensitivity to precise boundary detection, which is important in heterogeneous cancer tissues.

Inference time measures the average processing duration per slide (in seconds), evaluated on CPU for classical methods and GPU for GrandQC. This metric is essential to gauge scalability for high-volume TCGA cohorts, ensuring methods are feasible in clinical settings without excessive computational resources.

## 4. Results and Discussions

### 4.1. TCGA Dataset Results

For each slide, the predicted tissue mask *P* is compared to the ground-truth mask *G* after binarizing both to 0,1 and, if necessary, resizing the prediction to match the ground-truth resolution. Two overlap metrics are computed: mean Intersection over Union (mIoU), which penalizes both over- and under-segmentation, and mean Dice Class Consistency (mDCC), which assigns twice the weight to overlap regions to better handle class imbalance.

All slides in the evaluation set were down-sampled to 10 μm ${\mathrm{px}}^{-1}$ resolution, and masks were generated accordingly. We report mIoU, mDCC, and average per-slide inference time (in seconds). The classical methods (Otsu, K-Means, and Double-Pass) were executed on a 12-core CPU, while GrandQC was run on both CPU and an RTX-4090 GPU. GrandQC’s performance serves as an in-domain upper bound, given its training on similar TCGA data.

For each slide, we computed IoU and Dice against the ground-truth mask. Study-level mIoU/mDCC in Table 2 are simple arithmetic means over all 3322 slides. Inference time is the per-slide runtime averaged over the same 3322 slides.

Aggregate results, weighted by cohort slide counts, demonstrate GrandQC’s leadership in mIoU and mDCC with moderate GPU inference time. Our proposed Double-Pass, an annotation-free hybrid method, follows closely in accuracy while achieving the lowest CPU inference times among the high-performing methods, significantly outperforming K-Means in efficiency and Otsu in accuracy.

Table 3 provides a breakdown by cancer type, revealing performance variations across cohorts. GrandQC consistently achieves the highest mIoU and mDCC in all cohorts, attributable to its supervised training on annotated TCGA slides, which enables it to learn cohort-specific patterns like staining variations and tissue morphologies. In contrast, the annotation-free classical methods, particularly our Double-Pass, rely solely on unsupervised image statistics and heuristics, yet manage to attain competitive scores. For example, in LIHC, Double-Pass trails GrandQC closely in both mIoU and mDCC, while K-Means slightly edges it out in accuracy but at a much higher computational cost. Otsu, while generally the fastest among single classical methods, exhibits lower accuracy, particularly in complex cohorts like BRCA and GBM, where under-segmentation is more pronounced.

These per-cohort results underscore GrandQC’s superior performance due to its in-domain training, which equips it to handle diverse cancer-specific challenges, such as necrotic areas in GBM or sparse tissues in BRCA. However, the classical methods offer compelling trade-offs: they require no annotations or GPU resources, making them accessible for preliminary processing in computational resource-constrained settings. Notably, our Double-Pass stands out by striking an optimal balance, delivering mIoU and mDCC values very close to GrandQC in several cohorts, such as CHOL and HNSC, at consistently sub-0.3-second CPU inference times—faster than Otsu in many cases while surpassing it in accuracy across most cohorts, and dramatically quicker than K-Means without sacrificing much precision. This remarkable speed stems from its innovative hybrid design, which fuses complementary passes (color-based artifact rejection and efficient downsampled clustering) to mitigate the limitations of single classical approaches, avoiding the full-pixel clustering overhead seen in K-Means.

The efficiency of Double-Pass is particularly useful in digital pathology, where processing thousands of gigapixel WSIs from large cohorts like TCGA can become a bottleneck. Rapid inference on standard CPUs enables scalable preprocessing in high-volume labs, reducing overall analysis time, minimizing computational costs, and allowing pathologists and AI models to focus on clinically relevant regions without delays. This is especially important in resource-limited settings or during real-time applications, where GPU access may be unavailable, making Double-Pass a practical, high-impact solution for advancing cancer research and diagnostics.

Overall, the results highlight cohort-dependent variability, likely driven by differences in tissue density, staining intensity, and artifact prevalence. For instance, hepatic cohorts (CHOL and LIHC) yield higher scores across all methods due to denser, well-stained tissues, whereas central nervous system (GBM) and breast (BRCA) cohorts present greater challenges with sparse or heterogeneous regions. While GrandQC sets the accuracy benchmark, Double-Pass’s annotation-free nature, exceptional speed, and near-comparable segmentation quality position it as a standout alternative for scalability, effectively reducing preprocessing bottlenecks and enhancing workflow efficiency.

### 4.2. Qualitative Results

**Figure 2:**
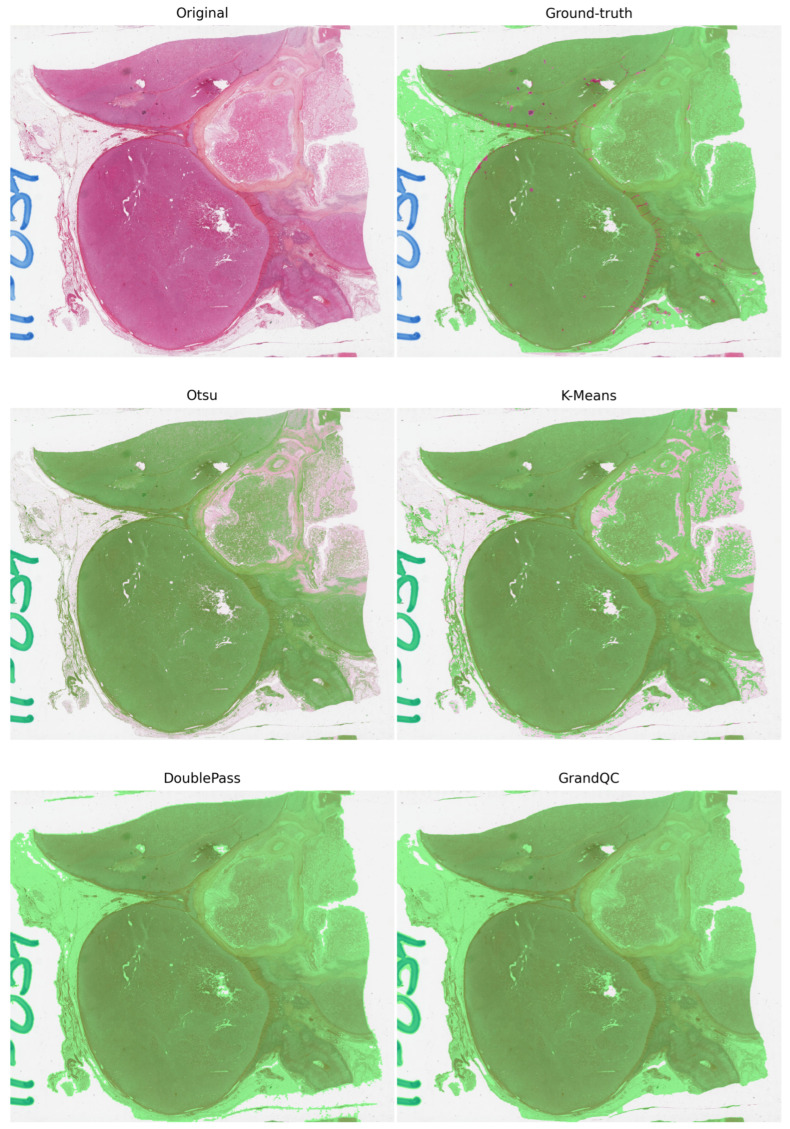
Visual comparison on an adrenocortical carcinoma (TCGA-ACC) thumbnail. Every panel shows the detector’s mask overlaid in transparent green; pink areas denote disagreements with the manual annotation. Otsu and Kmeans visibly returned more false-negative results than Double-Pass and GrandQC. The latter two had similar masks. Dice scores were as follows: Otsu 0.84, K-Means 0.90, Double-Pass 0.96, GrandQC 0.97.

**Figure 3:**
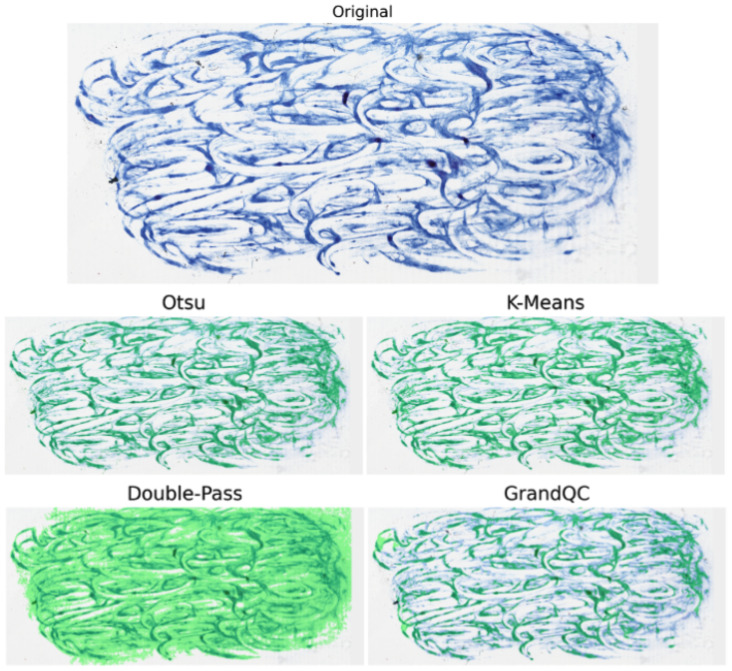
Detectors applied on Ziehl–Neelsen sputum smear (for tasks like detecting acid-fast bacilli). Starting from the original ZN image (blue counterstain background), we generated green overlays representing detection outputs from four approaches: Otsu global thresholding, K-Means color clustering, GrandQC quality-guided selection, and a Double-Pass refinement that re-screens uncertain margins. Although the first three methods mark many relevant regions, each omits patches that can contain acid-fast bacilli. The Double-Pass procedure expands coverage to include all such candidate areas, yet still suppresses large expanses of non-tissue signal, improving downstream sensitivity without a large specificity penalty.

**Figure 4:**
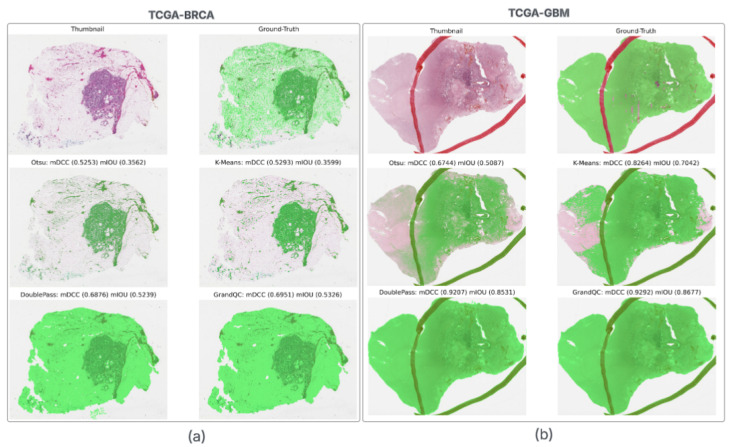
In (**a**), the breast cancer (BRCA) sample is composed mostly of a sparse tissue section with clusters of tumor cells in fibrous stroma. Otsu (mDCC 0.5253, mIOU 0.3562) and K-Means (mDCC 0.5293, mIOU 0.3599) overlook isolated nests, risking delayed detection of invasive components and inaccurate staging. Double-Pass (mDCC 0.6876, mIOU 0.5239) and GrandQC (mDCC 0.6951, mIOU 0.5326) provide more comprehensive coverage and avoid inaccurate staging. In (**b**), the glioblastoma (GBM), contains more pale, eosinophilic, irregular necrotic areas. Otsu’s thresholding (mDCC 0.6744, mIOU 0.5087) and K-Means (mDCC 0.8264, mIOU 0.7042) miss substantial necrotic regions, potentially leading to underestimation of tumor grade and misprognosis. Double-Pass (mDCC 0.9207, mIOU 0.8531) and GrandQC (mDCC 0.9292, mIOU 0.8677) better capture these features.

Qualitative comparisons (Figure 2, Figure 3 and Figure 4) illustrate Double-Pass’s smoother masks in cancer slides, minimizing missed regions.

These visualizations reveal that Double-Pass produces more contiguous masks with fewer false negatives compared to Otsu, particularly in heterogeneous tissues like ACC, where it achieves near-GrandQC quality without annotations. In non-cancer examples like sputum, methods show robustness to different stains, but cancer-specific findings indicate Double-Pass’s strength in avoiding over-inclusion of artifacts, enhancing downstream AI reliability. Limitations include potential misses in very faint areas; however, the hybrid approach mitigates this better than pure classical methods.

Double-Pass delivers deep learning–comparable accuracy while remaining lightweight and annotation-free, a key in pathology diagnosis where expert time is limited.

In cancer cohorts, performance varied: Higher scores in cohorts like LIHC, and lower scores in others like GBM. This highlights Double-Pass’s potential in diverse tissues. In BRCA, Double-Pass achieved a competitive performance. Limitations include the thumbnail resolution missing micro-details; future work could integrate multi-scale approaches.

## 5. Conclusions

In this study, we introduced Double-Pass, a novel, annotation-free hybrid method for efficient tissue detection in whole-slide images (WSIs). Benchmarked alongside classical Otsu and K-Means methods, as well as the deep learning-based GrandQC UNet++, Double-Pass was evaluated on over 3000 annotated TCGA slides spanning nine distinct cancer cohorts. It achieved a strong performance, with a mean Intersection over Union (mIoU) of 0.826 and an average CPU inference time of 0.203 s per slide, offering a compelling balance between segmentation accuracy and computational speed, without the need for GPU acceleration or manual annotations. In contrast to deep learning models, which require labor-intensive annotations and significant computing resources, Double-Pass combines two complementary classical strategies—gray pixel filtering and color-based clustering—to enhance robustness and detect tissue regions even in heterogeneous or faintly stained cancer slides. This design enables annotation-free segmentation that is both efficient and scalable, making it well suited for high-throughput digital pathology workflows, particularly in resource-limited environments. Furthermore, we demonstrated the broader applicability of Double-Pass through qualitative results on Ziehl–Neelsen-stained slides used in tuberculosis diagnosis. Its ability to generalize across both oncological and non-oncological tissue types highlights its flexibility as a preprocessing tool. By fusing the speed and accessibility of classical methods with segmentation quality approaching that of deep learning, Double-Pass addresses the limitations of existing approaches and emerges as a practical, lightweight solution for large-scale WSI analysis, annotation-free training pipelines, and foundational model development in computational pathology.

To contextualize our findings, we note that operating at 10 μm ${\mathrm{px}}^{-1}$ trades granularity for throughput and can miss thin slivers or detached micro-islands. Another limitation is that the pipeline outputs a binary tissue–background mask and does not subclass artifacts (e.g., pen marks, blur), so residual false positives may persist. Evaluation relied only on TCGA data from GrandQC masks, so broader external validation across scanners and stains would further test generalizability. These constraints motivate further research.

Looking ahead, future directions could leverage the strengths of annotation-free methods like Double-Pass to enhance training foundational models in digital pathology. Based on its proven efficiency (0.203 s/slide on CPU) and accuracy (mIoU 0.826) approaching supervised benchmarks across TCGA cohorts, integrating Double-Pass into self-supervised pre-training pipelines—such as those for models like pathology-adapted DINOv2—could enable automatic masking of non-tissue regions in large unlabelled WSI datasets, covering more cancer-relevant features without annotations. This promises improved model generalization and downstream performance on tasks like tumor segmentation and prognosis prediction, as empirical studies could confirm through comparisons on diverse cancer types, advancing scalable computational pathology workflows.

## Figures and Tables

**Table 1:** Cancer types covered in the study.

TCGA Code	Disease Type	Cancer Primary Site
ACC	Adenomas and Adenocarcinomas	Adrenal Gland
BRCA	9 Cancer Types	Breast
CESC	SCC and Adenocarcinoma	Cervix
CHOL	Cholangiocarcinoma	Biliary/Liver
DLBC	Diffuse Large B-cell Lymphoma	Lymph Nodes
ESCA	SCC and Adenocarcinoma	Esophagus
GBM	Glioblastoma Multiforme	Central Nervous System
HNSC	Squamous Cell Carcinoma	Head and Neck
LIHC	Adenomas and Adenocarcinomas	Liver

**Table 2:** Summary of characteristics and performance metrics for all benchmarked tissue detection methods.

Method	Method Type	Annotations Required?	mIoU	mDCC	CPU Time (s)	GPU Time (s)
Otsu (TIAToolbox)	Classical thresholding	None	0.779	0.861	0.294	–
K-Means (MSTD)	Unsupervised clustering	None	0.840	0.899	2.487	–
Double-Pass (ours)	Hybrid (colour intensity)	None	0.826	0.895	0.203	–
GrandQC (CPU)	Supervised deep model	Yes	0.871	0.924	2.431	–
GrandQC (GPU)	Supervised deep model	Yes	0.871	0.924	–	0.580

**Table 3:** Tissue detection performance per cancer type.

TCGA Code	Cancer Primary Site	Images	Method	mIoU	mDCC	Time (s)
TCGA-ACC	Adrenal Gland	206	Otsu	0.857	0.921	0.400
			K-Means	0.912	0.953	9.391
			Double-Pass (ours)	0.891	0.941	0.251
			GrandQC (GPU)	0.921	0.958	0.717
			GrandQC (CPU)	0.921	0.958	2.851
TCGA-BRCA	Breast	1100	Otsu	0.668	0.786	0.273
			K-Means	0.738	0.834	1.368
			Double-Pass (ours)	0.818	0.894	0.215
			GrandQC (GPU)	0.844	0.910	0.585
			GrandQC (CPU)	0.844	0.910	2.482
TCGA-CESC	Cervix	279	Otsu	0.827	0.895	0.217
			K-Means	0.874	0.924	1.615
			Double-Pass (ours)	0.779	0.860	0.184
			GrandQC (GPU)	0.855	0.912	0.552
			GrandQC (CPU)	0.855	0.912	2.161
TCGA-CHOL	Biliary/Liver	39	Otsu	0.938	0.967	0.330
			K-Means	0.977	0.988	10.162
			Double-Pass (ours)	0.950	0.974	0.258
			GrandQC (GPU)	0.970	0.985	0.749
			GrandQC (CPU)	0.970	0.985	3.053
TCGA-DLBC	Lymph Nodes	42	Otsu	0.812	0.874	0.174
			K-Means	0.849	0.897	5.902
			Double-Pass (ours)	0.798	0.865	0.171
			GrandQC (GPU)	0.852	0.899	0.532
			GrandQC (CPU)	0.852	0.899	2.090
TCGA-ESCA	Esophagus	155	Otsu	0.826	0.896	0.155
			K-Means	0.894	0.936	8.301
			Double-Pass (ours)	0.846	0.909	0.197
			GrandQC (GPU)	0.900	0.942	0.552
			GrandQC (CPU)	0.900	0.942	2.259
TCGA-GBM	Central Nervous System	860	Otsu	0.796	0.871	0.332
			K-Means	0.859	0.909	1.294
			Double-Pass (ours)	0.784	0.863	0.188
			GrandQC (GPU)	0.848	0.905	0.549
			GrandQC (CPU)	0.848	0.905	2.363
TCGA-HNSC	Head and Neck	274	Otsu	0.821	0.891	0.268
			K-Means	0.894	0.935	1.356
			Double-Pass (ours)	0.855	0.914	0.199
			GrandQC (GPU)	0.904	0.945	0.589
			GrandQC (CPU)	0.904	0.945	2.568
TCGA-LIHC	Liver	367	Otsu	0.914	0.951	0.350
			K-Means	0.953	0.972	2.610
			Double-Pass (ours)	0.907	0.949	0.194
			GrandQC (GPU)	0.946	0.971	0.578
			GrandQC (CPU)	0.946	0.971	2.352

## Data Availability

Data from GrandQC and TCGA are publicly available. The Python implementations of the algorithms described in this paper, including the Double-Pass method, are available at https://github.com/aimas-upb/efficient-tissue-detection-wsi (accessed on 31 August 2025).

## Abbreviations

    The following abbreviations are used in this manuscript:

WSI	Whole-Slide Image
TCGA	The Cancer Genome Atlas
mIoU	Mean Intersection over Union
mDCC	Mean Dice Class Consistency
SCC	Squamous cell carcinoma.
CNN	Convolutional neural network.

## Appendix A. Pseudocode for Detection Methods

Here, we provide pseudocode for the four methods in a narrative style suitable for medical doctors and practitioners. These are annotation-free (except GrandQC) and run at thumbnail level for efficiency in cancer WSIs.

**Algorithm A1** Global Otsu Tissue Mask
**Input:** Thumbnail *I* ∈ $R^{H\times W\times 3}$ **Output:** Mask $M\in {\left\{0,255\right\}}^{H\times W}$ 1: **function** GlobalOtsu(*I*) 2: G←RGB2Gray(I) ▹ convert to grayscale 3: $T^{★}\leftarrow OtsuThreshold\left(G\right)$ ▹ compute optimal threshold 4: $M\leftarrow 255\times 1_{\left\{G<T^{★}\right\}}$ ▹$M_{i,j}=255$ if $G_{i,j}<T^{★}$, else 0 5: **return** *M* 6: **end function**

**Algorithm A2** Colour-Statistics K-Means Tissue Mask
**Input:** RGB thumbnail *I* ∈ $R^{H\times W\times 3}$ **Output:** Mask $M\in {\left\{0,255\right\}}^{H\times W}$ 1: **function** ColourKMeans(*I*) 2: X←Reshape(I,(H · W,3)); scale to [0,1] ▹ reshape to (H · W)×3 and normalise 3: μ←Mean(X,axis=1) ▹ mean brightness 4: σ←StdDev(X,axis=1) ▹ RGB variability 5: F←Stack([μ,σ]) ▹ 2D feature matrix; N=H · W rows 6: C←KMeans(F,K=2) ▹ cluster into 2 groups 7: $k^{★}\leftarrow \arg\min_{k}\;{\mathrm{mean}}_{i\in k}\mu_{i}$ ▹ choose darker cluster 8: $M\leftarrow 255\times 1_{\left\{C=k^{★}\right\}}$ ▹H×W mask: $M_{i,j}=255$ if pixel (i,j) is in cluster $k^{★}$, else 0 9: M←Close(M,5); M←Open(M,5) ▹ morphological smoothing 10: **return** *M* 11: **end function**

**Algorithm A3** Double-Pass Tissue Detector
- **Input:** RGB thumbnail *I* ∈ $R^{H\times W\times 3}$ - **Output:** Mask $M\in {\left\{0,255\right\}}^{H\times W}$ 1: **function** FilterGrays(*I*) 2: J←Sharpen(I) ▹ enhance edges 3: M←¬(|R−G|≤15∧|R−B|≤15∧|G−B|≤15) ▹ non-grey pixel detection 4: M←Dilate(M,5); M←Close(M,5) ▹fill gaps & smooth 5: M←RemoveSmall(M,5000) ▹ remove small objects 6: **return** $M_{\mathrm{FG}}\leftarrow M$ 7: **end function** 8: **function** DownKMeans(*I*) 9: $I_{↓}\leftarrow Resize\left(I,0.25\right)$ downsample to 25% 10: $X\leftarrow Reshape(I_{↓},\left(-1,3\right))$ ▹ reshape downsampled image 11: C←KMeans(X,K=2) ▹cluster into 2 groups 12: $k^{★}\leftarrow \arg\min_{k}\;{\mathrm{mean}}_{i\in k}{\mathrm{intensity}}_{i}$ ▹ choose darker cluster 13: $M_{↓}\leftarrow 1_{\left\{C=k^{★}\right\}}$ ▹binary mask: ${\left(M_{↓}\right)}_{i,j}=1$ if pixel in cluster $k^{★}$, else 0 14: $M\leftarrow Resize(M_{↓},\left(H,W\right))$ ▹upsample to original size 15: M←Close(M,5); M←Open(M,5) ▹ smooth mask 16: **return** $M_{\mathrm{KM}}\leftarrow M$ 17: **end function** 18: **function** Double-Pass(*I*) 19: $M_{\mathrm{FG}}\leftarrow FilterGrays\left(I\right)$ ▹ first pass: non-grey detection 20: $M_{\mathrm{KM}}\leftarrow DownKMeans\left(I\right)$ second pass: colour clustering 21: $M\leftarrow M_{\mathrm{FG}}\vee M_{\mathrm{KM}}$ ▹combine masks 22: M←Close(M,5); M←Open(M,5) ▹smooth final mask 23: **return** 255×M ▹convert to 0/255 mask 24: **end function**

**Algorithm A4** GrandQC Inference Pipeline
- **Input:** RGB thumbnail *I* ∈ $R^{H\times W\times 3}$ - **Output:** Mask $M\in {\left\{0,255\right\}}^{H\times W}$ 1: **function** GrandQC(*I*) 2: J←JPEG(I,Q=80) ▹ compress thumbnail 3: $\left\{\left(P_{j},o_{j}\right)\right\}\leftarrow Tile\left(J,512\right)$ ▹ split into 512×512 patches 4: **for all** tiles $\left(P_{j},o_{j}\right)$ **do** 5: $N_{j}\leftarrow Norm_{\mathrm{ImageNet}}\left(P_{j}\right)$ ▹ normalise patch 6: ${\hat{P}}_{j}\leftarrow UNet++\left(N_{j},\mathrm{amp}=1\right)$ ▹predict tissue probabilities 7: $L_{j}\leftarrow \arg\;\max\left({\hat{P}}_{j},\;\mathrm{axis}=\mathrm{class}\right)$ ▹ select most likely class 8: Paste$\left(\hat{L},\;L_{j},\;o_{j}\right)$ ▹insert $L_{j}$ into $\hat{L}$ at offset $o_{j}$ 9: **end for** 10: $M\leftarrow 255\times 1_{\left\{\hat{L}=0\right\}}$ ▹set $M_{i,j}=255$ if ${\hat{L}}_{i,j}=0$, else 0 11: **return** *M* 12: **end function**

## References

[1] Weng Z., Seper A., Pryalukhin A., Mairinger F., Wickenhauser C., Bauer M., Glamann L., Blaker H., Lingscheidt T., Hulla W. (2024). GrandQC: A comprehensive solution to quality-control problem in digital pathology. Nat. Commun..

[2] Otsu N. (1979). A threshold selection method from gray-level histograms. IEEE Trans. Syst. Man Cybern..

[3] Song Y., Cisternino F., Mekke J.M., de Borst G.J., de Kleijn D.P.V., Pasterkamp G., Vink A., Glastonbury C.A., van der Laan S.W., Miller C.L. (2023). An automatic entropy method to efficiently mask histology whole-slide images. Sci. Rep..

[4] Chen P., Yang L. (2019). Tissueloc: Whole-slide digital pathology image tissue localization. J. Open Source Softw..

[5] Bándi P., Balkenhol M., van Ginneken B., van der Laak J., Litjens G. (2019). Resolution-agnostic tissue segmentation in whole-slide histopathology images with convolutional neural networks. PeerJ.

[6] Wang D., Khosla A., Gargeya R., Irshad H., Beck A.H. (2016). Deep learning for identifying metastatic breast cancer. arXiv.

[7] Pocock J., Graham S., Vu Q.D., Jahanifar M., Deshpande S., Hadjigeorghiou G., Shephard A., Bashir R.M.S., Bilal M., Lu W. (2022). TIAToolbox as an end-to-end library for advanced tissue image analytics. Commun. Med..

[8] Filiot A., Jacob P., Kain A.M., Saillard C. (2024). Phikon-v2, a large and public feature extractor for biomarker prediction. arXiv.

[9] Vorontsov E., Bozkurt A., Casson A., Shaikovski G., Zelechowski M., Liu S., Severson K., Zimmermann E., Hall J., Tenenholtz N. (2024). Virchow: A million-slide digital pathology foundation model. arXiv.

[10] Ceachi B., Cioplea M., Mustatea P., Dcruz J.G., Zurac S., Cauni V., Popp C., Mogodici C., Sticlaru L., Cioroianu A. (2024). A new method of artificial-intelligence-based automatic identification of lymphovascular invasion in urothelial carcinomas. Diagnostics.

[11] Zurac S., Mogodici C., Poncu T., Trăscău M., Popp C., Nichita L., Cioplea M., Ceachi B., Sticlaru L., Cioroianu A. (2022). A new artificial-intelligence-based method for identifying Mycobacterium tuberculosis in Ziehl–Neelsen stain on tissue. Diagnostics.

[12] Bándi P., van de Loo R., Intezar M., Geijs D., Ciompi F., van Ginneken B., van der Laak J., Litjens G. (2017). Comparison of different methods for tissue segmentation in histopathological whole-slide images. arXiv.

[13] Riasatian A., Rasoolijaberi M., Babaei M., Tizhoosh H.R. (2020). A comparative study of U-Net topologies for background removal in histopathology images. arXiv.

[14] Marczyk M., Wrobel A., Merta J., Polanska J. Post-processing of thresholding or deep learning methods for enhanced tissue segmentation of whole-slide histopathological images. Proceedings of the 12th International Conference on Bioimaging.

[15] Nechaev D., Pchelnikov A., Ivanova E. (2025). HISTAI: An open-source, large-scale whole-slide image dataset for computational pathology. arXiv.

[16] Patil A., Jain G., Diwakar H., Sawant J., Bameta T., Rane S., Sethi A. (2025). Semantic segmentation based quality control of histopathology whole-slide images. arXiv.

[17] Nicke T., Schacherer D., Schäfer J.R., Artysh N., Prasse A., Homeyer A., Schenk A., Höfener H., Lotz J. (2025). Tissue Concepts v2: A supervised foundation model for whole-slide images. arXiv.

